# Posaconazole exposure in critically ill ICU patients: a need for action

**DOI:** 10.1007/s15010-023-02078-9

**Published:** 2023-07-27

**Authors:** Christina König, Melanie Göpfert, Stefan Kluge, Dominic Wichmann

**Affiliations:** Department of Intensive Care Medicine, University Medical Center Hamburg-Eppendorf, Martinistraße 42, 20251 Hamburg, Germany

**Keywords:** Posaconazole, Therapeutic drug monitoring, Invasive aspergillosis, Critically ill patients

## Abstract

**Purpose:**

Posaconazole is an antifungal drug currently being used for prophylaxis and treatment of invasive fungal infections such as aspergillosis. To date, therapeutic drug monitoring (TDM) of posaconazole is recommended with the use of oral suspension, but the potential need of TDM with the use of IV formulations is rising. Therefore, we aimed to investigate the pharmacokinetics of IV posaconazole in critically ill patients.

**Methods:**

In a prospective study, we analysed 168 consecutivelly collected posaconazole levels from 10 critically ill patients drawn during a 7 day curse. Posaconazole concentrations were measured using a chromatographic method. Demographic and laboratory data were collected, and the data was analysed using descriptive statistics.

**Results:**

We included 168 posaconazole levels, resulting in a median trough of 0.62 [0.29–1.05] mg/L with 58% not reaching the suggested target of 0.5 mg/L for fungal prophylaxis. Moreover, 74% of the trough levels were under the target of 1 mg/L which is proposed for the treatment of aspergillosis.

**Conclusion:**

Posaconazole exposure is highly variable in critically ill patients resulting in potentially insufficient drug concentrations in many cases. TDM is highly recommended to identify and avoid underexposure.

**Trial registration number:**

NCT05275179, March 11, 2022.

## Introduction

In addition to patients with haematological malignancies, who represent the classical risk group for invasive aspergillosis, critically ill patients treated in intensive care units (ICU) have been identified as a population at risk in recent years [1, 2]. Amongst others, liver diseases, chronic obstructive lung disease, diabetes mellitus and ongoing treatment with immunosuppressive drugs like steroids or acute viral pneumonia have proven to be promoting factors [3]. The latter became particularly evident in the context of the current SARS-CoV-2 pandemic, where the risk factors for a severe course of COVID-19 showed large overlaps with the risk factors for invasive aspergillosis [4, 5]. In addition, specific therapies with dexamethasone and tocilizumab contribute to the problem. For the treatment of invasive aspergillosis, current guidelines from international societies recommend initial therapy with voriconazole or isavuconazole [6, 7]. In a recent trial, posaconazole demonstrated non-inferiority to voriconazole making it a suitable treatment option for invasive aspergillosis [8].

Due to a higher burden of organ dysfunctions, ICU patients are in general receiving a variety of medications for their underlying disease. In the majority of cases, oral medication is applied by an enteral feeding tube which is potentially undermining the superiority of the tablet galenic as it is only allowing administration of the oral suspension. The latter is generally showing a limited and food-dependent bioavailability compared to the gastro-resistant tablets [9, 10]. Therefore, intravenous (IV) posaconazole is often a more suitable option for ICU patients. With azoles inhibiting the cytochrome P450 enzymes but also being substrate to these enzymes (e.g. isavuconazole), potential drug–drug interactions are of particular concern in this cohort [11]. Posaconazole itself shows limited hepatic metabolism and is mainly excreted by bile as the unchanged drug [12]. However, hypoalbuminemia is a potential factor influencing posaconazole exposure as it is highly protein-bound (> 98%) [13]. In addition to this, uridine diphosphate-glucuronosyltransferases and P-glycoprotein are involved in the metabolization of posaconazole. Interindividually, both enzymes show a large number of polymorphisms resulting in different metabolization rates [14]. Posaconazole shows a half-life elimination of approximately 15–35 h. Therefore, an initial loading dose is needed prior to the maintenance dose in order to achieve sufficient drug exposure [15].

Non-attainment of drug levels such as reported for voriconazole is associated with an increased risk of break through infections [16, 17]. Therefore, therapeutic drug monitoring (TDM) is required and highly recommended for voriconazole [6, 7]. Additionally, TDM for posaconazole is recommended by some societies aiming for trough concentrations (*C*_min_) of 0.5 to 0.7 mg/L for prophylaxis and > 1 mg/L for the treatment of invasive aspergillosis [7]. With oral posaconazole, reaching the target level is highly dependent on the galenic of the formulation. The oral absorption of the suspension is known to be highly variable [18]. To improve this, a delayed-release tablet was developed [19]. But even with this, non-target attainment in patients on oral medication is of great concern [20]. Despite the known difficulties, a study was recently published assessing the use of the oral solution in critically ill patients and confirmed the known issues [21]. Still, systematic data of intravenous (IV) posaconazole exposure in critically ill patients are lacking to date. Therefore, our study aimed to generate prospective data on the pharmacokinetic (PK) of IV posaconazole in critically ill patients.

## Materials and methods

### Study population

The study was conducted in the department of intensive care medicine the University Medical Center Hamburg-Eppendorf, Germany. During the study period, the department comprised 11 ICUs with 128 beds and served all specialties of adult intensive care medicine. Adult critically ill patients with an indication for posaconazole were eligible for the study after written consent from the patient or their legal representative. The study was approved by the ethics committee of the Hamburg Chamber of Physicians (Ref.: PV7263), complied with the Declaration of Helsinki and was registered at Clinicaltrials.gov (NCT05275179). Patient data were collected from the department’s electronical patient data management system (PDMS, Integrated Care Manager® (ICM), Version 9.1—Draeger Medical, Luebeck, Germany). The data included gender, age, body mass index, comorbidities, admission diagnosis, organ support (mechanical ventilation, vasopressor support and dialysis), laboratory test results, sequential organ failure assessment score (SOFA) [22] and simplified acute physiology score (SAPS) [23]. Data management and descriptive analysis were performed with Excel (Version 16.62 – Microsoft Corporation, 2022).

### Therapeutic drug monitoring of posaconazole

Posaconazole was administered via a central line according to the manufacturer’s recommendation at a dose of 300 mg dissolved in 250 ml 0.9% normal saline and subsequently administered via gravity-mediated infusion over 90 min once daily. On day one, an additional loading dose of 300 mg IV was administered. Blood samples (2.5 mL) were taken 1 h (peak), 4 h (transit) and 23 h (trough) after the infusion for 7 consecutive days. Samples were centrifuged for 10 min at 3,000 rpm and stored at – 70 °C until further analysis. Total drug concentrations were measured by high-performance liquid chromatography with fluorescence detection using a validated commercial kit from ChromSystemS^®^ (München, Germany).

### Pharmacokinetic analysis

The PK parameters, such as clearance (CL), half-lives (*t*_1/2_) and volume of distribution (*V*_d_) of posaconazole, were determined using the Eqs. (1–4) and performed within Excel (Version 16.62- Microsoft Corporation, 2022). Trough and peak concentrations during the observation period were observed values. Visualisation was performed using Prism (GraphPad, Version 9, San Diego, CA, USA).

1 $${\text{CL}} \left[ {L/h} \right] = \frac{{\ln 2 \times V_{d} \left[ L \right]}}{t1/2\left[ h \right]}$$

2 $$t1/2\left[ h \right] = \frac{\ln 2}{{ke}}$$

3 $$ke = \frac{{\ln \left( {c_{1} /c_{2} } \right)}}{\Delta t}$$

4 $$V_{{\text{d}}} = \frac{{{\text{dose}}}}{{C_{\max } }},$$

*c*_1_ is the concentration on time point 1 [mg/L], *c*_2_ is the concentration on time point 2 [mg/L], *C*_max_ is the peak concentration [mg/L], *CL* clearance [L/h], dose: posaconazole in [mg], *ke* is the constant of elimination, *t*_1/2_ is the half-life [h], *V*_d_ volume of distribution [L], Δt is the time between time points 1 and 2

## Results

During the study period, from February 1st 2021 to January 31st 2022, ten patients (9 male) were included. The median age was 64 years (IQR 54–68), the median body mass index 25.9 kg/m^2^ (IQR 22.1–27.7), the median SOFA score 10.5 (IQR 8.3–12.8) and median SAPS score 50 (IQR 48.5–53.8). Eight patients received posaconazole for therapy of a probable aspergillosis (classified by the criteria of the European Organization for Research and Treatment of Cancer) [24] and 2 in prophylactic indication. Risk factors for aspergillosis were malignant haematological disease or allogeneic stem cell transplantation in 9 cases (7 myeloid and 2 lymphatic malignancies) and 1 case of non-small cell lung cancer. Six of the patients with haematological malignancies had received allogeneic stem cell transplantation. The reasons for the ICU admission were septic shock (*n* = 4), respiratory failure (*n* = 4), renal failure (*n* = 1) and encephalopathy (*n* = 1). Eight patients received invasive mechanical ventilation, 7 vasopressor therapy and 6 renal replacement therapy with continuous veno-venous haemodialysis (CVVHD) at a mean dialysis dose of 30 mL/kg body weight per hour. Three patients died during the study period. More data are presented in Table 1. In total, 168 posaconazole levels were analysed, with 55 and 59 being trough and maximum concentrations, respectively. Median trough concentrations were 0.62 [0.29–1.05] mg/L (see Table 2). A biphasic elimination could be observed for posaconazole (alpha: 1–4 h; beta 4–23 h post infusion). Half-lives (*t*_1/2_) were markedly different and presented with a median of 12.6 h for the alpha (*t*_1/2 α_) phase 29.5 h for *t*_1/2β_. Total body clearance (CL) was 13 L/h for this patient cohort.

**Table 1:** Demographic patient data

Characteristic	Value
Male (*n*)	9
Age [years]	64 [54–68]
Height [cm]	180 [172–180]
Weight [kg]	82 [72–89]
Body mass index [kg/m^2^]	25.9 [22.1–27.7]
SAPS	50 [48.5-–53.8]
SOFA Score	10.5 [8.3–12.8]
Invasive ventilation (*n*)	8
Vasopressor use (*n*)	7
CVVHD (*n*)	6
C-Reactive protein [mg/dL]	120 [50–230]
Leucocyte count [Mrd/L]	2.7 [0.5–6.2]
Thrombocyte count [Mrd/dL]	32 [13–63]
Haemoglobin [g/gL]	7.7 [7.2–8]
Haematocrit [%]	22 [21–23]
International normalised ratio [%]	85 [59–96]
Albumin [mg/dL]	18 [16–20]
Aspartate aminotransferase [U/l]	43 [19–293]
Alanine aminotransferase [U/l]	29 [16–91]
γ-Glutamyltransferase [U/l]	240 [101–350]
Alkaline phosphatase [U/l]	241 [192–295]
Glutamate dehydrogenase [U/l]	25 [2–94]
Bilirubin [mg/dl]	1.3 [0.8–7.3]

Data are presented as medians, interquartile ranges [] or numbers

*SAPS* simplified acute physiology score, *SOFA* sequential organ failure assessment score, *CVVHD* continuous veno-venous hemodialysis

**Table 2:** Posaconazole data

Characteristic	Value
Samples total (*n*)	168
Trough levels (*n*)	55
Peak level (*n*)	59
*C*_min_ [mg/L]	0.62 [0.29–1.05]
*C*_max_ [mg/L]	1.56 [1.15–2.0]
Posaconazole dose [mg/d]	300
*t*_1/2_ [h]	19.4 [8.7–29.8]
*t*_1/2 α_ [h]	12.6 [6.7–20.4]
*t*_1/2β_ [h]	29.5 [11.6–45.4]
CL [L/h]	13 [7.8–20.74]
CL_α_ [L/h]	20.8 [11.8–30.4]
CL_β_ [L/h]	9.4 [4.4–16.9]
Overall target attainment	
*C*_min_ ≥ 0.5 mg/L (%)	58
*C*_min_ ≥ 0.7 mg/L (%)	40
*C*_min_ ≥ 1.0 mg/L (%)	26

Data are presented as medians, interquartile ranges [] or numbers

*C*_*max*_ peak level, *C*_*min*_ trough level, *CL* clearance, *t*_*1/2*_ half-life

**Fig. 1:**
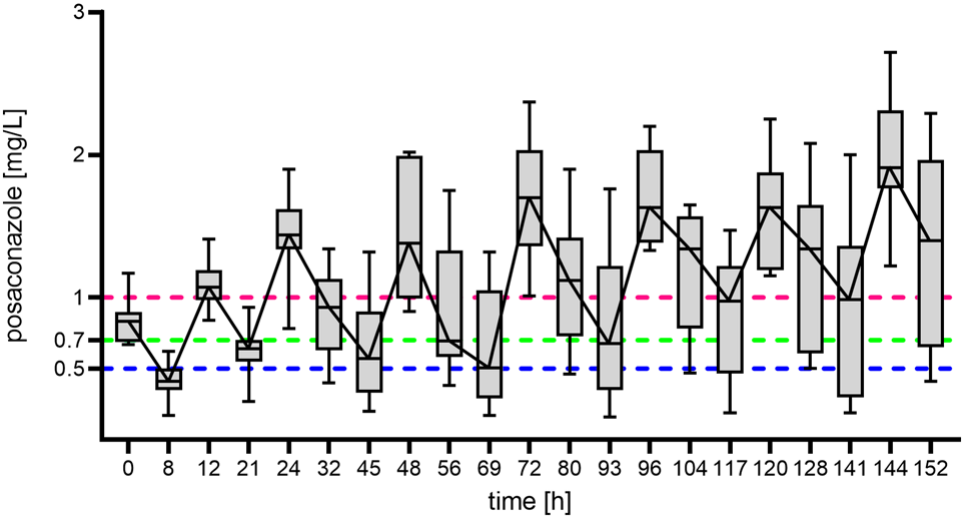
Posaconazole concentration time curves during the first week of treatment. Values are shown as linked medians with 75% quartiles (grey boxes). The horizontal lines indicate the postulated target values (prophylaxis: blue line = 0.5 mg/L and green line = 0.7 mg/L, therapy: purple line = 1.0 mg/L)

Moreover, 42% and 60% of the observed trough levels did not achieve the moderate or high target for prophylaxis (0.5 mg/L and 0.7 mg/L), respectively. The target discussed for aspergillosis therapy (1 mg/L) was not reached in 74% of the patients. The exposure of posaconazole in the overall cohort is shown in Fig. 1, with relatively low trough concentrations after the loading dose (until 21 and 45 h) and a relative plateau after approximately 117 h. The moderate target of  ≥ 0.5 mg/L for prophylaxis and ≥ 1 mg/L for therapy is only achieved in 80% and 0% of the patients after the completion of the loading dose (day 2 eq. 21 h). After day 6 (= 141 h), trough levels of ≥ 0.5 mg/L, ≥ 0.7 mg/L and ≥ 1.0 mg/L could be achieved in 67%, 67% and 44% of the included patients, respectively.

## Discussion

In this study, posaconazole exposure was first described in critically ill patients with an extended sampling regimen resulting in a PK profile for posaconazole of 7 days. In 8 patients, posaconazole was used as treatment of a probable invasive aspergillosis in the remaining two in a prophylactic indication. Most authors including Ullmann et al. [7] set a target trough concentration of 0.7 mg/L for prophylaxis. Whereby, other meta-analyses [25] conclude that a more moderate target trough concentration of 0.5 mg/L is sufficient for prophylaxis. In our cohort of critically ill patients, only 40% of all trough levels reached the 0.7 mg/L target, and even the 0.5 mg/L target was attained in only 58%. Moreover, the threshold for treatment (1 mg/L) was achieved in only 26% of all the obtained samples. This incidence is quite low, compared to previous studies such as by Van Daele et al. [26] where target attainment was achieved in approximately 70% of ECMO patients. In addition, target attainment (1 mg/L) was reported by Cornely et al. [27] in about 70% of healthy volunteers. Total body CL of 13 (7.8–20.74) L/h for posaconazole in our cohort was within the reported ranges of 16.8 (11.1–21.7) L/h [28] and 8.7 (8–10.6) L/h [26, 29]. None of the patients showed high posaconazole levels greater than 3.25 mg/L which might be associated with toxicity [7].

It should be noted that the PK/PD target for posaconazole efficacy is still a matter of debate. As supposed by haematological guidelines [30] and the European Society of Clinical Microbiology and Infectious Diseases [7], a trough level above 0.7 mg/L is associated with a lower risk of breakthrough infections when used for fungal prophylaxis. Weighting this against the risk of potential drug toxicity other authors [25] favour a target of 0.5 mg/L. Moreover, when used for treatment purposes, a value of 1.0 mg/L is recommended for invasive aspergillosis [7]. On the other hand, therapeutic success is also defined by a total AUC_0–24_/MIC of 167–178. Regarding the susceptibility breakpoint of 0.125 mg/L for *Aspergillus* species, a target AUC_0–24_ of 20.9–22.5 mg*h/L would be suitable [31]. Regardless of the relevant target parameter to be defined, our work shows that pharmacokinetic data from regulatory studies do not reflect the reality in critically ill patients [32]. This is evident by the fact that after the application of the loading dose, only 80 to 30% of patients had sufficient drug exposure for fungal prophylaxis (0.5–0.7 mg/L), and none of the patients would have been treated optimal (1.0 mg/L). Despite the fact that the loading dose was reliably administered, steady concentrations occurred only after 5 days of therapy (see Fig. 1). Similar observations but with a slightly better target attainment for prophylaxis have been shown by van Daele and colleagues [29].

Also, by the end of the study period (day 6) when steady-state conditions were reached, only 67% and 42% had optimal drug exposure for either prophylaxis (0.7 mg/L) or treatment (> 1 mg/L) of invasive mould infections. Despite the high data density of our study, the low number of patients is a limiting factor when it comes to generalisation. The majority of our patients had an underlying haematological disease. Although the conditions leading to intensive therapy (homeostasis disorders, septic shock, renal or pulmonary failure) are identical, the results shown here may not be fully transferable to other (e.g. surgical) patient populations.

Since critical illness is associated with many pathophysiological changes which may lead to adapted dosing regimens, this PK data is of interest to the intensivists and consultant infectious disease specialists. Posaconazole’s major elimination pathway is through biliary secretion of the unchanged parent compound with neglectable renal clearance of a glucuronide conjugate [12]. Therefore, posaconazole PK is unlikely to be affected by renal insufficiency or receiving continuous renal replacement therapies (CRRT) as in our study cohort (*n* = 6) [13]. However, there is still controversy using cyclodextrin-based formulations in patients with renal insufficiency or renal replacement therapies due to its potential to accumulate [33]. Contrary, studies have shown that the cyclodextrin used in intravenous posaconazole formulations will be efficiently eliminated via haemodialysis [34]. Therefore, the use of intravenous posaconazole was based on individual risk–benefit considerations. Moreover, as posaconazole being a highly protein-bound (98%) drug, hypoalbuminemia may affect posaconazole elimination and exposure. As we only measured total posaconazole concentrations and our patient cohort presented with hypoalbuminemia (median albumin level of 18 g/dL), the free and therefore active fraction might have been higher [35]. Thus, further studies are needed to quantify the effect of hypoalbuminemia on free and total posaconazole concentrations to fully understand its PK. Due to the small number of only 10 patients in this study, it is difficult to conclusively evaluate the data. Consequently, a more detailed analysis such as population pharmacokinetic modelling should be performed to further evaluate the variability in posaconazole exposure.

## Conclusions

Posaconazole exposure in critically ill patients is different compared to non-critically ill haematological patients. A high proportion of patients did neither achieve prophylactic nor therapeutic concentrations throughout the observational period. Therefore, TDM is highly recommended in critically ill patients to attain appropriate drug concentrations and to avoid breakthrough infections and therapeutic failure.

## Data Availability

Data may be obtained from the authors upon request on the basis of the European General Data Protection Regulations.
